# Complete Genome Sequence of *Ornithogalum* Mosaic Virus Infecting *Gladiolus* spp. in South Korea

**DOI:** 10.1128/genomeA.00816-16

**Published:** 2016-08-11

**Authors:** Sang-Yun Cho, Seungmo Lim, Hongsup Kim, Seung-In Yi, Jae Sun Moon

**Affiliations:** a Seed Testing & Research Center, Korea Seed & Variety Service, Gimcheon, Republic of Korea; b Molecular Biofarming Research Center, Korea Research Institute of Bioscience and Biotechnology, Daejeon, Republic of Korea; c Biosystems and Bioengineering Program, University of Science and Technology, Daejeon, Republic of Korea

## Abstract

We report here the first complete genome sequence of *Ornithogalum* mosaic virus (OrMV) isolated from Taean, South Korea, in 2011, which was obtained by next-generation sequencing and Sanger sequencing. The sequence information provided here may serve as a potential reference for other OrMV isolates.

## GENOME ANNOUNCEMENT

*Gladiolus* (family *Iridaceae*) is a genus of perennial cormous flowering plants, and its cultivation may be affected by various diseases, causing reduced yield and quality of flowers (1). *Ornithogalum* mosaic virus (OrMV), a member of the *Potyvirus* genus in the *Potyviridae* family, causes severe leaf mosaic symptoms as well as flower deformation in *Ornithogalum* species and can cause severe disease in some ornamentals (2). To date, a small number of full-genome sequences of OrMV isolates, all of them sequenced from orchids, are available in GenBank (accession numbers NC_019409, JQ807995, JQ807996, and JQ807997) (3–5).

Here, we report the complete genome sequence of the OrMV isolate Taean from the Chungcheongnam-do province of South Korea, which was isolated in June 2011 from leaf stripes of *Gladiolus* species. Total RNA was extracted from these symptomatic leaves using TRI reagent (Molecular Research Center, OH). To identify the potential causative agent, the extracted total RNA was used to synthesize cDNA, followed by screening using the next-generation sequencing Illumina HiSeq 2500 platform (Theragen Etex Bio Institute, Suwon, South Korea) (6). Raw sequencing data were processed using SeqGenesis (Daejeon, South Korea). OrMV isolate Taean was detected from a *de novo* assembly of five contigs of lengths 2,726 bases, 2,374 bases, 1,414 bases, 1,714 bases, and 924 bases. PCR was carried out to amplify the virus genome, except for both ends, based on the sequences of the contigs, using AccuPower ProFi Taq PCR PreMix (Bioneer, Daejeon, South Korea). Rapid amplification of 5′ and 3′ cDNA ends (RACE) was performed using the 5′-/3′-RACE system (Invitrogen, CA), according to the manufacturer’s instructions. All PCR products were cloned into the T&A cloning vector (RBC Bioscience, Taipei, Taiwan), and the sequences of the clones were analyzed by Macrogen (Daejeon, South Korea).

The complete genome sequence of this new isolate, Taean, is 9,475 nucleotides (nt) in length, and it shares 75% identity and 86% query coverage with OrMV isolate SW3.1 (GenBank accession no. JQ807995), isolated from an orchid in Australia. In the 5′-untranslated region (UTR), a 36-nt insertion was observed compared to that in the reported isolates. One open reading frame, flanked by the 5′- and 3′-UTRs, was identified by the DNAMAN 5.0 program (Lynnon Biosoft, Quebec, Canada) (7).

This is the first report of the full-length sequence of OrMV derived from *Gladiolus* species. This virus can potentially be transmitted to different perennial cormous flowering plants of the iris family.

### Accession number(s).

The complete genome sequence of OrMV isolate Taean has been deposited in GenBank under the accession no. KU981083.
